# An explainable machine learning approach using contemporary UNOS data to identify patients who fail to bridge to heart transplantation

**DOI:** 10.3389/fcvm.2024.1383800

**Published:** 2024-05-20

**Authors:** Mamoun T. Mardini, Chen Bai, Maisara Bledsoe, Benjamin Shickel, Mohammad A. Al-Ani

**Affiliations:** ^1^ Department of Health Outcomes and Biomedical Informatics, College of Medicine, University of Florida, Gainesville, FL, United States; ^2^ Division of Cardiovascular Medicine, University of Florida, Gainesville, FL, United States

**Keywords:** heart transplantation, machine learning, UNOS, intra-aortic balloon pump, Impella

## Abstract

**Background:**

The use of Intra-aortic Balloon Pump (IABP) and Impella devices as a bridge to heart transplantation (HTx) has increased significantly in recent times. This study aimed to create and validate an explainable machine learning (ML) model that can predict the failure of status two listings and identify the clinical features that significantly impact this outcome.

**Methods:**

We used the UNOS registry database to identify HTx candidates listed as UNOS Status 2 between 2018 and 2022 and supported with either Impella (5.0 or 5.5) or IABP. We used the eXtreme Gradient Boosting (XGBoost) algorithm to build and validate ML models. We developed two models: (1) a comprehensive model that included all patients in our cohort and (2) separate models designed for each of the 11 UNOS regions.

**Results:**

We analyzed data from 4,178 patients listed as Status 2. Out of them, 12% had primary outcomes indicating Status 2 failure. Our ML models were based on 19 variables from the UNOS data. The comprehensive model had an area under the curve (AUC) of 0.71 (±0.03), with a range between 0.44 (±0.08) and 0.74 (±0.01) across different regions. The models' specificity ranged from 0.75 to 0.96. The top five most important predictors were the number of inotropes, creatinine, sodium, BMI, and blood group.

**Conclusion:**

Using ML is clinically valuable for highlighting patients at risk, enabling healthcare providers to offer intensified monitoring, optimization, and care escalation selectively.

## 1 Introduction

Temporary mechanical circulatory support (tMCS) enables clinicians to stabilize cardiogenic shock patients until HTx (1, 2). Since the 2018 heart allocation update, tMCS utilization has tripled (3). Intra-aortic balloon pump (IABP) or Impella (5.0 and, more recently, 5.5 versions) is used in approximately half of heart transplantation patients. These devices significantly differ in hemodynamic effects, vascular access, and complication risk profile. The majority of IABP and Impella evidence comes from the population of acute coronary syndrome or peri-cardiac intervention use. Identifying the suitable device for the right patient at the right time to achieve optimal pre and post-HTx outcomes remains a formidable task and a knowledge gap (4).

In the current healthcare landscape, precision medicine is gaining momentum, and machine learning (ML) is proving to be a valuable resource for clinicians to understand intricate relationships between hemodynamics demographics, tMCS, and medical history and the dynamics of HTx listing practices. Previous studies have investigated the application of ML on United Network for Organ Sharing (UNOS) data for predicting post-heart transplant mortality (5–7) and survival on waiting lists (8). However, the reported model performances ranged from an AUC of 0.5–0.7, indicating the challenges associated with the complex nature of the data and patient characteristics highlighted by the heterogeneous clinical phenotypes, high acuity, and rapidly developing tMCS.

It is worth noting that many of the ML models were developed using data from before the 2018 heart allocation update was implemented and at a time when mechanical circulatory support was rarely used and linked to unfavorable outcomes. This study aims to employ explainable ML methods to rank and weigh the clinical factors determining the failure of status two listings. Developing and optimizing such models is vital for the upcoming continuous distribution heart transplant system to be adaptable to demographic and practice changes, unlike its predecessors (9).

## 2 Methods

### 2.1 Study population

We utilized the UNOS registry to identify heart transplant candidates listed between 2018 and 2022 as UNOS Status 2 and supported with Impella (5.0 or 5.5) or intra-aortic balloon pump. The local institutional review board approved the study, and informed consent was waived due to minimal risk to participants.

### 2.2 Features description

*Sociodemographic features* included age at the time of listing, gender, race, body mass index, and height. BMI and height were included as they are essential determinants of waitlist time and have implications on anatomic suitability to certain tMCS devices. We split Race into five categories: Asians, Black, Hispanic, White, and others. *Lifestyle and habits features* included only smoking history. *Biological characteristics* included blood groups categorized into A, B, AB, and O. *Medical devices and treatment features* included tMCS device, implanted defibrillator, mechanical ventilation, dialysis (intermittent hemodialysis and continuous renal replacement therapy), and multiorgan transplantation (Heart and Liver, Heart and Kidney). *Clinical hemodynamics,* measured within 24 h prior to MCS*,* included pulmonary capillary occlusion pressure, cardiac index, resting heart rate, and pulmonary artery pulsatility index (PAPi), which was calculated as the pulmonary artery pulse pressure divided by the right atrial pressure. *Biochemistry features,* measured within 24 h prior to MCS, included creatinine, aspartate aminotransferase, total bilirubin, albumin, sodium, and international normalized ratio. *Medication and drug administration features* included whether the patient was on antiarrhythmics, vasopressin, dopamine, dobutamine, epinephrine, milrinone, and the number of inotropes. *Geographic features* included the UNOS region. UNOS divides the United States into 11 transplant regions. The purpose of these regions is to ensure a balance between the availability of organs and the number of people waiting for transplants in any given area. We have included the region in model derivation to account for the wide variation in tMCS utilization between regions (4, 10). We excluded region 6 due to the small number of patients (<150 patients). The *primary outcome* is the failure of tMCS, which encompasses various scenarios where the device fails to keep the patient in a stable enough condition to receive a heart transplant. It is defined as death while on the waiting list, being too sick to transplant, being listed as an inactive patient due to a high risk of transplantation (Status 7) or upgrading to UNOS status 1.

### 2.3 Data preprocessing

We considered patient characteristics significantly different between the two groups (patients successfully transplanted while on Status 2 vs. patients who failed to transplant) as the input features for ML models (Table 1). To eliminate the highly correlated features from hemodynamic and biochemical measurements, PAPi was created by using pulmonary systolic pressure (PASP), pulmonary dynamic pressure (PADP), and central venous pressure (CVP). Thus, PASP, PADP, and CVP were removed from the data. Additionally, we found that mean artery pressure is highly correlated with pulmonary capillary occlusion pressure, and blood urea nitrogen is highly correlated with creatinine. Therefore, blood urea nitrogen and mean artery pressure were subsequently removed. For each patient, we created two missing value indicators to check if the patient had any missing values in the hemodynamic and biochemical measurements. In total, 19 features were included to train and evaluate the ML models. Categorical features were encoded into numerical values and handled directly by the ML algorithm.

**Table 1:** Comparison of patients’ characteristics between patients who were successfully transplanted during status 2 and those who failed to transplant.

Variable	Failure *N* = 504)	Non-failure (*N* = 3,674)	*p*-value
AGE (SD)	52.3 (13.6)	54.3 (12.37)	**<0**.**001**
Body mass index (SD)	27.95 (4.95)	27.17 (4.87)	**<0**.**001**
Height, years (SD)	175.9 (10.2)	174.32 (9.8)	**<0** **.** **001**
Female (%)	96 (19.0%)	885 (24.1%)	**0**.**014**
Race (%)			**<0** **.** **001**
Asian	26 (5.2%)	141 (3.8%)	
Black	178 (35.3%)	1,007 (27.4%)	
White	249 (49.4%)	2,075 (56.5%)	
Hispanic	44 (8.7%)	412 (11.2%)	
Other	7 (1.4%)	39 (1.1%)	
Smoking (%)	188 (37.3%)	1,449 (39.4%)	0.382
Blood group (%)			**<0** **.** **001**
A	141 (28.0%)	1,386 (37.7%)	** **
AB	20 (4.0%)	134 (3.6%)	** **
B	77 (15.3%)	595 (16.2%)	** **
O	266 (52.8%)	1,559 (42.4%)	** **
Device (%)			**<0** **.** **001**
IABP	389 (77.2%)	3,139 (85.4%)	** **
Impella	115 (22.8%)	535 (14.6%)	
Implemented defibrillator (%)	352 (69.8%)	2,524 (68.7%)	0.639
On mechanical ventilation (%)	13 (2.6%)	21 (0.6%)	**<0** **.** **001**
On dialysis (%)	24 (4.8%)	120 (3.3%)	0.111
Diabetes mellitus (%)	158 (31.3%)	1,124 (30.6%)	0.769
Cardiomyopathy			0.558
Ischemic	133 (26.4%)	921 (25.1%)	
Non-ischemic	371 (73.6%)	2,753 (74.9%)	
Multiorgan transplantation			
Heart and liver (%)	7 (1.4%)	46 (1.3%)	0.964
Heart and kidney (%)	31 (6.2%)	348 (9.5%)	**0**.**019**
Hemodynamics			
Pulmonary capillary occlusion pressure, mmHg (SD)	25.9 (8.0)	24.4 (7.8)	**0**.**004**
Cardiac index, L/min/m^2^ (SD)	1.9 (0.5)	1.9 (0.6)	0.309
Resting heart rate, beats/min (SD)	99.4 (19.1)	89.7 (19.0)	**0**.**001**
Pulmonary artery pulsatility index (SD)	2.7 (2.5)	3.2 (3.5)	**0**.**020**
Biochemistry			
Creatinine, mg/dl (SD)	1.8 (1.7)	1.5 (1.3)	**0**.**001**
Aspartate aminotransferase, IU/L (SD)	43.4 (109.1)	40.9 (94.7)	0.709
Total bilirubin, mg/dl (SD)	1.3 (1.5)	1.2 (1.2)	0.149
Albumin, g/dl (SD)	3.6 (0.5)	3.7 (0.5)	**0**.**002**
Sodium, mmol/L (SD)	134.0 (5.2)	135.2 (4.2)	**<0** **.** **001**
International normalized ratio	1.5 (0.5)	1.4 (0.4)	**0**.**018**
On antiarrhythmics (%)	253 (50.2%)	1,639 (44.6%)	**0**.**021**
On dopamine (%)	53 (10.5%)	182 (5.0%)	**<0** **.** **001**
On vasopressin (%)	65 (12.9%)	76 (2.0%)	**<0** **.** **001**
On epinephrine (%)	94 (18.7%)	209 (5.7%)	**<0** **.** **001**
On milrinone (%)	353 (70.0%)	2,239 (60.9%)	**<0** **.** **001**
UNOS region (%)			**<0** **.** **001**
1	27 (5.4%)	140 (3.8%)	
2	61 (12.1%)	341 (9.3%)	
3	84 (16.7%)	455 (12.4%)	
4	55 (10.9%)	378 (10.3%)	
5	48 (9.5%)	575 (15.7%)	
6	17 (3.4%)	55 (1.5%)	
7	34 (6.7%)	351 (9.6%)	
8	27 (5.4%)	263 (7.2%)	
9	47 (9.3%)	304 (8.3%)	
10	35 (6.9%)	301 (8.2%)	
11	69 (13.7%)	511 (13.9%)	
On antiarrhythmics (%)	253 (50.2%)	1,639 (44.6%)	**0** **.** **021**
Number of inotropes (SD)	1.1 (0.8)	0.7 (0.6)	**<0** **.** **001**
Missing value indicator			
Missing any hemodynamic measurements (%)	319 (63.3%)	2,432 (66.2%)	0.216
Missing any biochemical measurements (%)	423 (83.9%)	2,919 (79.5%)	0.022

Bold values indicate statistically significant.

### 2.4 Machine learning modeling

We applied eXtreme Gradient Boosting (XGBoost) to build the ML models. XGBoost is an ensemble learning algorithm based on decision trees in which models are developed sequentially to increase the performance of the prior trees by using gradient descent to minimize errors (11). We developed two types of models: (1) a comprehensive model that included all patients in our cohort and (2) distinct models designed for each of the 11 UNOS regions. For region-specific models, we only considered regions with a minimum of 150 patients, excluding region 6.

### 2.5 Handling missing and imbalanced data

We have taken several measures to deal with missing data and class imbalance in our dataset and chose the one that enhanced the performance of our ML model. To impute missing data, we examined four methods: (1) mean imputation, where missing values are replaced with the feature's mean value; (2) median imputation, where missing values are replaced with the feature's median value; (3) K-nearest neighbor imputation, which predicts and fills in missing data based on the similarity of k-nearest data points, and (4) XGBoost's built-in imputation mechanism, which utilizes gradient boosting to handle missing values in a way that reduces prediction errors. For counteracting data imbalance, we explored three methods: under-sampling, Synthetic Minority Oversampling Technique (SMOTE), and assigning increased weights to the minority classes during the training process. Under-sampling reduces the number of majority class samples, SMOTE generates synthetic samples for the minority class, and assigning increased weights to the minority classes during training gives more importance to the minority class samples. Supplementary Table S1 shows the best combination of data imputation and data resampling strategies determined for each model.

### 2.6 Model performance and evaluation

We evaluated the performance of our models by conducting internal validation to ensure rigor and enhance confidence in model generalizability. We utilized a 5 × 5 nested cross-validation (CV) approach consisting of inner and outer loops where data is divided into folds. In each outer fold, one-fifth of the patient's records were an independent test set, and the rest (four-fifths) were a training set. The outer training set was then equally split into five inner folds served as an independent validation set, and the other four folds served as an inner training set (inner loop). The inner loop is responsible for model training and hyperparameter tuning (the process of searching for the optimal combination of hyperparameters of the model). In contrast, the outer loop is responsible for error estimation and generalization. We used grid search for hyperparameter tuning, in which exhaustive combinations of the chosen hyperparameters were applied to train the models. The average value and standard deviation of the area under the curve (AUC), accuracy, balanced accuracy, sensitivity, and specificity from the five outer folds were calculated and reported. The data preprocessing, imputation, and grid search steps were implemented using the Python Sklearn package. The XGBoost algorithm was implemented using the XGBoost package.

### 2.7 Model interpretation & feature ranking

We used the SHapley Additive exPlanation (SHAP) to interpret the trained ML models. SHAP is a model-agnostic explanation technique that is commonly used to interpret the results from the ML model. We generated the SHAP summary plot to visualize the importance and association between each feature and the outcome. The association is represented using a sign and a magnitude. The sign of the SHAP value indicates the directionality of the association between the corresponding feature and the outcome (e.g., a positive SHAP value indicates that the related feature contributes to a higher risk of transplant failure while on status 2). The magnitude of the SHAP value indicates the relative contribution of the prediction. We computed the SHAP value for all the patients in the test set in each outer fold. After five iterations (five outer folds), each patient was assigned a SHAP value for each feature. We developed a heatmap summarizing the rank of features across models trained for different UNOS regions (12, 13).

### 2.8 Statistical analysis

We compared the characteristics of patients successfully transplanted during Status 2 and those who failed to transplant. Two sample *t*-test was used to compare the numerical characteristics that are normally distributed, while the Wilcoxon rank sum test was used to compare the numerical characteristics that are not normally distributed. We used the Chi-square test to examine the independence of categorical characteristics between the two groups. The significance level was set at *p* < 0.05. Statistical analyses were done using the open-source package *scipy* in Python.

## 3 Results

In our study, we analyzed data from 4,178 patients listed as Status 2. Among them, 12% experienced primary outcomes indicating Status 2 failure. Impella 5.0 or 5.5 was used in 15.6% (650 patients) of the cohort, while the remainder were supported with IABP. Table 1 compares demographic, clinical, and biochemical characteristics between the failure group (*N* = 504) and the non-failure group (*N* = 3,674). Several variables such as age, BMI, height, race, blood group (A, B, AB, or O), device type used (IABP vs. Impella), sodium, vasoactive medications, and UNOS region (11 regions) show statistically significant differences between the two groups. Notably, the failure group was slightly younger and had higher BMI, had lower creatinine, albumin, sodium, and INR, and had a different distribution of blood groups and vasoactive medications used.

The ML model's performance in predicting the primary outcome (status 2 failure) across various UNOS regions is outlined in Table 2. The area under the curve (AUC) of the comprehensive model was 0.71 (±0.03) for all regions, with a range between 0.44 (±0.08) and 0.74 (±0.01). The models' specificity (survival on Status 2) ranged between (0.75–0.96). The accuracy varies by region, with the highest accuracy of 0.90 being achieved in Region 5 and the lowest accuracy of 0.69 being observed in Region 4. The table also shows the balanced accuracy, a more nuanced measure when classes are imbalanced. The balanced accuracy scores were generally lower across all regions, with an overall value of 0.65. The AUC suggests moderate predictive power at 0.71 overall, but this metric also shows regional variations. The sensitivity scores were notably low across all regions. On the other hand, specificity scores were consistently high, indicating good performance in identifying true negatives (success on Status 2).

**Table 2:** Performance metrics of predicting failure on transplant of status 2 using XGBoost across different UNOS regions.

Region	Accuracy	Balanced accuracy	AUC	Sensitivity	Specificity
All patients	0.72 (0.02)	0.65 (0.02)	0.71 (0.03)	0.54 (0.04)	0.75 (0.02)
1	0.74 (0.02)	0.56 (0.09)	0.60 (0.11)	0.30 (0.25)	0.82 (0.08)
2	0.79 (0.03)	0.65 (0.06)	0.71 (0.04)	0.44 (0.10)	0.85 (0.02)
3	0.76 (0.01)	0.54 (0.04)	0.59 (0.05)	0.21 (0.08)	0.86 (0.01)
4	0.69 (0.07)	0.45 (0.06)	0.44 (0.08)	0.13 (0.07)	0.77 (0.07)
5	0.90 (0.05)	0.56 (0.06)	0.72 (0.08)	0.17 (0.11)	0.96 (0.05)
7	0.85 (0.02)	0.51 (0.03)	0.60 (0.08)	0.09 (0.07)	0.93 (0.02)
8	0.86 (0.01)	0.55 (0.07)	0.69 (0.06)	0.16 (0.15)	0.93 (0.02)
9	0.82 (0.06)	0.61 (0.10)	0.72 (0.11)	0.32 (0.18)	0.89 (0.06)
10	0.86 (0.02)	0.53 (0.03)	0.59 (0.10)	0.11 (0.06)	0.94 (0.02)
11	0.78 (0.05)	0.65 (0.07)	0.74 (0.01)	0.49 (0.17)	0.82 (0.07)

Each value is the mean and standard deviation of the 5-fold nested cross-validation.

**Figure 1:**
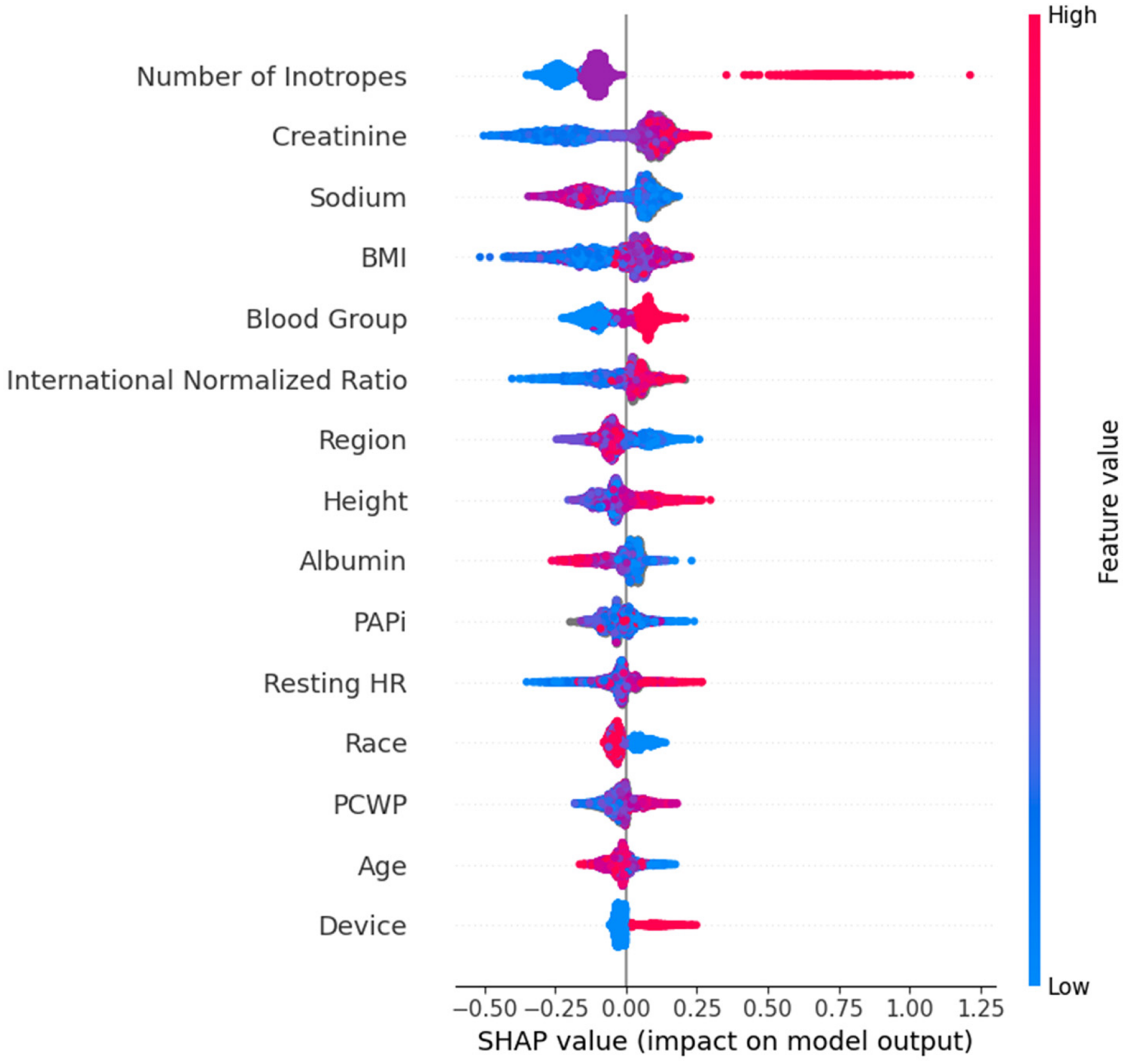
The rank of the top 15 most essential features in predicting status 2 failure. The X-axis represents the SHAP values dichotomized into two regions: positive SHAP values (>0) on the right side and negative SHAP values (<0) on the left side. Dots on the right side represent positive contributions toward predicting status 2 failure. While dots on the left side represent a negative contribution toward predicting status 2 failure. For continuous features (e.g., Creatinine), the color ranges from blue to red, indicating low to high contribution to the outcome. For categorical features [e.g., Multi-organ (Kidney)], red dots signify the presence of the condition (e.g., the patient has a kidney transplant), whereas blue dots indicate its absence.

**Figure 2:**
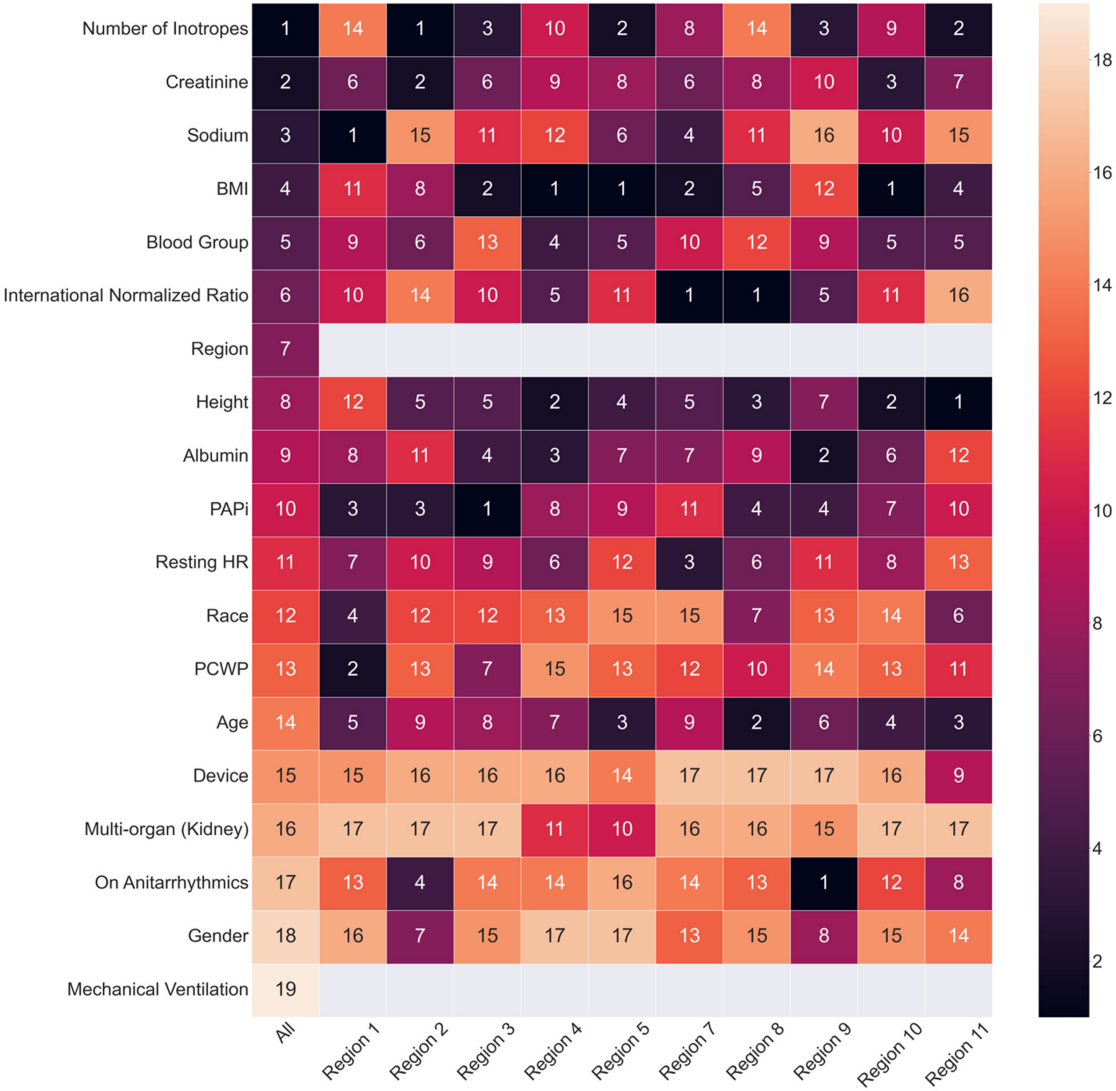
Heatmap of feature ranking generated from XGBoost trained across UNOS regions. The features are ordered by the ranking from the comprehensive XGBoost model. The numbers indicate the rank of the feature importance for each cohort. The grey blocks indicate that the region and mechanical ventilation are not used as input features for machine learning models built on separate UNOS regions.

The SHAP ranking, illustrated in Figure 1, shows the top 15 most essential features in predicting Status 2 failure. We found that the lead outcome determinants were sodium (region 1), the number of inotropes (region 2, overall), PAPi (region 3), BMI (region 4, 5, 10), international normalized ratio (INR) (region 7, 8), on antiarrhythmics (region 9), height (region 11). The number of inotropes, creatinine, sodium, BMI, and blood group were the top five most important predictors in the overall model. The number of inotropes has the highest impact on predicting status 2 failure; higher numbers increase the likelihood of status 2 failure. Similarly, elevated creatinine levels increase the risk of status 2 failure. Sodium, BMI, Blood Group, INR, Region, Height, and Albumin seem to have a neutral impact on predicting the outcome. The concentration of heart resting points suggests that higher values could be associated with increased risk in some patients. PAPi, race, PCWP, and age are centered around zero SHAP value, indicating a lower influence on predicting the outcome. The device ranked 15th among all included variables (out of 19), indicating lower importance. Figure 2 shows the heatmap summarizing the rank of features across models trained for different UNOS regions. Finally, Supplementary Table S2 shows the device utilization ratio (# IABP: # Impella) and the ratio between the number of patients successfully bridging to transplant and those who failed across regions.

## 4 Discussion

In this study, we aimed to use explainable ML methods to develop and validate a data-driven model that predicts the failure of status 2 listing and ranks the clinical features that have the most impact on the primary outcome. The latter function is vital as it provides patients and care teams with actionable targets to address so they can improve the outcomes of tMCS or specific reasons to consider an alternative BTT strategy. Our results show that the specificity of the ML models was consistently high, indicating good performance in identifying true negatives (success on Status 2). There is, however, significant regional variability in feature ranking, which indicates that such ML methods need to be tuned to accommodate not only the patient and the machine but also the HTx practice context. While the models seem adept at identifying BTT failure (high specificity), it has limited ability to confidently assure that those predicted to do well until transplantation will indeed remain stable (low sensitivity). Despite this shortcoming, the current ML model proves clinically helpful in highlighting patients at risk so that intensified monitoring, optimization, and care escalation can be selectively lamented.

The most predictive features vary across regions, and notable patterns emerge. The number of inotropes consistently appeared important in predicting status 2 failure in most regions. A higher number indicates greater disease severity and contributes positively to the risk of status 2 failure. Elevated serum creatinine and International Normalized Ratio (INR) indicate that poor kidney and liver function reserves are also major outcome determinants. BMI also appeared in many regions, suggesting the role of both obesity and malnutrition as influencing factors. Further investigation is needed to understand whether weight affects BTT outcomes due to intrinsic patient factors or by affecting waitlist time. The role of race is highlighted in many areas, which suggests the potential for racial disparities in healthcare utilization. Finally, the significance of age and blood group were not uniform between regions, indicating that these factors can be mitigated by optimizing practice patterns.

Contrary to expectations, the type of tMCS device (IABP vs. Impella) was less significant than patient-related factors in predicting Status 2 failure. However, it shows that using Impella can increase the risk of failure to bridge to transplantation, which is consistent with the current stream of evidence that IABP-supported patients fare better than most other status 2 listed patients (14, 15). Impella provides robust circulatory support for the left ventricle, with maximal blood flow over 5 L/min. However, this benefit is often reduced by some degree of aortic regurgitation and decreased LV preload (16). On the other hand, the IABP requires smaller vascular access (8 Fr vs. 23 Fr) and has a variable hemodynamic response that depends on vascular stiffness, body size, and the ability of the left ventricle to augment function in response to afterload reduction [8]. In reality, most status 2 patients require partial left ventricular unloading, and the transplant community continues to lack any pragmatic prospective study in comparing IABP vs. Impella, specifically as BTT. The available data from post-acute coronary syndrome state is not translatable to a population with a high prevalence of acute on chronic systolic failure that are likely to utilize the device for weeks rather than hours or days.

A few studies have explored the potential of ML for predicting post-heart transplant mortality (5–7) and survival on waiting lists (8) using UNOS data. These models, with AUC values ranging from 0.5 to 0.7, highlight the complexities associated with the data and the diversity of patient demographics. Notably, a significant portion of these models was based on data that predates the 2018 heart allocation update. Additionally, during that time, mechanical circulatory support was still in its early stages and often resulted in suboptimal outcomes. Our study addressed this knowledge gap by using explainable ML methods and focusing on contemporary data, especially after the 2018 heart allocation update, to create a more refined predictive model. Our work aimed to overcome the limitations of previous models and provided a better understanding of the factors that influence transplant outcomes in today's medical landscape. A direction to include explainable ML models in a continuously learning national transplant system will allow continued data feed for model training and lead to optimized performance that matches the current state of the HTx practice environment.

This innovative approach naturally comes with several limitations that any adopter of these results must understand. First, the model is trained on a dataset with high missing rate that has inherent variability in reporting. Second, the model does not reflect variation between different health systems within each region. Third, the performance parameters were derived using 5-fold cross validation from the same dataset, which is less ideal than external validation. However, external validation was not possible and is not necessarily relevant because the model is fitted explicitly to a region and practice era. Ideally, resting this model will require prospective testing of a locally optimized version of the ML model to guide decisions and prove its effect on outcomes.

## 5 Conclusion

ML XGboost model can identify UNOS status 2 patients at high risk of deterioration while on tMCS with high specificity and limited sensitivity. This is an innovative approach to selecting the right tMCS for the right patient, identifying targets for intensified patient monitoring and optimization guided by the model's feature selection and developing an adaptive and continuously learning heart transplant system.

## Data Availability

The data analyzed in this study is subject to the following licenses/restrictions: Data available to researchers through a request form. Requests to access these datasets should be directed to Organ Procurement and Transplantation Network - OPTN.
