# Supplementary material for: An explainable machine learning approach using contemporary UNOS data to identify patients who fail to bridge to heart transplantation
Source: Front Cardiovasc Med. 2024 May 20;11:1383800. doi: 10.3389/fcvm.2024.1383800 (PMC11144884; doi:10.3389/fcvm.2024.1383800)
Supplement: Supplementary file 1 [file Datasheet1.pdf]

Supplementary Material

1 Supplementary Figures and Tables

1.1 Supplementary Tables

Supplementary Table 1. The best combination of data imputation and data resampling strategies determined for each model.

Region	Data Imputation Technique	Data Resampling Technique
All patients	Internal mechanism	Under sampling
1	KNN imputation	Under sampling
2	Median imputation	Weights
3	KNN imputation	SMOTE
4	Mean imputation	SMOTE
5	Mean imputation	Weights
7	Internal mechanism	Under sampling
8	Internal mechanism	Weights
9	Mean imputation	SMOTE
10	Mean imputation	SMOTE
11	Median imputation	Under sampling

Supplementary Table 2. Device utilization ratio (# IABP : # Impella) and the ratio between the number of patients successfully bridging to transplant and of those who failed across regions.

Region	Device Utilization Ratio	# Success/#Failure
All patients	5.4	7.3
1	7.3	5.2
2	4.2	5.6
3	3.2	5.4
4	2.7	6.9
5	3.9	12.0
7	15.0	10.3
8	21.3	9.7
9	14.3	6.5
10	8.6	8.6
11	8.4	7.4
